# Flame‐Retardant Ionogel Enabled by Lignin Molecular Networks for Fire Rescue

**DOI:** 10.1002/advs.202506901

**Published:** 2025-06-25

**Authors:** Zewei Ye, Haomeng Yu, Hongxia Xie, Wenwen Zhu, Shitao Shi, Chencong Liu, Yuanyuan Wang, Jiaqi Liao, Qingfeng Sun, Dawei Zhao, Xiaoping Shen

**Affiliations:** ^1^ College of Chemistry and Materials Engineering Zhejiang A&F University Hangzhou 311300 P. R. China; ^2^ Key Laboratory on Resources Chemicals and Materials of Ministry of Education Shenyang University of Chemical Technology Shenyang 110142 P. R. China

**Keywords:** flame‐retardant ionogel, harsh environments, intelligent applications, real‐time monitoring, wearable sensors

## Abstract

Ionogel intended for fire environments encounters considerable challenges stemming from intrinsic limitations, such as inadequate heat resistance and potential damage to their structural networks. Here, a glycol lignin ionogel (GLI) with integrated flame‐retardant, waterproof, and sensing capabilities for smart firefighting auxiliary equipment is presented. The ionogel, synthesized from the molecular network construction of Gly‐lignin, 1‐butyl‐3‐vinylimidazole tetrafluoroborate (BVIMBF_4_), and benzyl methacrylate (BZMA), forms a stable and elastic 3D conductive network through both chemical and physical crosslinking. This structure imparts remarkable mechanical properties, including considerable adhesion (30.4 kPa on flame‐retardant gloves), tensile strength (5.8 MPa), and high ionic conductivity. Notably, through the formation of a protective carbonized/B₂O₃ layer and chemical barrier gases during combustion, the ionogel exhibits excellent flame retardancy, with a weight loss of 41.8% at 800 °C. Additionally, GLI achieves real‐time thermal sensing via its negative temperature coefficient (NTC) behavior (TCR = −0.58% °C^−1^). Even after exposure to fire, GLI retains its temperature and motion‐sensing capabilities. Moreover, GLI demonstrates favorable biocompatibility and environmental degradability, broadening its applicability in sustainable and wearable electronics. The work provides new insights for the preparation of the next generation of fireproof multifunctional intelligent sensors.

## Graphical abstract

Wearable sensors for harsh, high‐temperature, fire‐prone environments face challenges in heat resistance and stability. This study introduces a flame‐retardant, waterproof glycol lignin ionogel (GLI) with a robust 3D network, exceptional tensile strength, strong adhesion, biocompatibility, eco‐safety, and dual temperature‐motion sensing, even after combustion, providing a solution for intelligent firefighting.

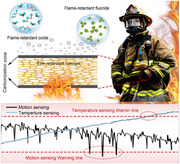

## 1 Introduction

Fire has played a pivotal role in human evolution, driving significant societal advancements.^[^ ^1^ ^]^ However, uncontrolled fires can lead to catastrophic disasters, resulting in irreparable loss of life, widespread property damage, and severe environmental consequences. Despite progress in firefighting technologies, fires continue to pose considerable risks, particularly to firefighters who are frequently exposed to dangerous environments during rescue operations. To reduce these risks, the development of functional materials for smart firefighting sensors has become increasingly crucial, enabling real‐time monitoring of environmental and physiological parameters. These materials can detect temperature fluctuations and track motion, providing early warnings to prevent injuries caused by overheating or other hazardous conditions.^[^ ^2^ ^]^

Ionogels, consisting of ionic liquids confined within polymeric or supramolecular networks,^[^ ^3^ ^]^ have gained considerable attention as soft, multifunctional materials for flexible sensing.^[^ ^4^ ^]^ Their unique combination of high ionic conductivity,^[^ ^5^ ^]^ thermal responsiveness,^[^ ^6^ ^]^ and mechanical compliance enables^[^ ^7^ ^]^ a wide range of functionalities, including strain and motion detection,^[^ ^8^ ^]^ temperature monitoring,^[^ ^9^ ^]^ pH^[^ ^10^ ^]^ and humidity sensing,^[^ ^11^ ^]^ etc. These sensing behaviors arise from distinct underlying mechanisms. For mechanical sensing, ionogels typically respond to external deformation through piezoresistive effects,^[^ ^12^ ^]^ capacitive changes,^[^ ^13^ ^]^ or stress‐induced rearrangement of ion transport channels,^[^ ^14^ ^]^ which collectively modulate the gel's electrical properties Temperature sensing, by contrast, is generally achieved via thermally induced variations in ionic mobility, impedance, or conductivity, with representative mechanisms including the negative temperature coefficient (NTC) effect^[^ ^15^ ^]^ and thermoelectric responses.^[^ ^16^ ^]^ The integration of these functionalities is strongly influenced by the synergistic interplay between polymer network topology, ionic liquid composition, and crosslinking strategy.^[^ ^17^ ^]^

With temperature sensing playing a pivotal role in early fire detection, recent studies have explored the application of ionogels in extreme environments (e.g., fire‐relevant scenarios) by enhancing their thermal responsiveness and reliability. Notably, Yinan Zhao^[^ ^18^ ^]^ synthesized a phosphorus‐containing ionic liquid‐based ionogel via UV‐initiated polymerization using acrylamide, low‐melting‐point poly anionic sodium polystyrene sulfonate, and poly cationic poly (diallyldimethylammonium chloride) as raw materials. The resulting ionogel demonstrated excellent ionic conductivity (1.33 S·m^−1^), imparting the material with precise temperature sensing and highly sensitive fire alarm capabilities. However, this system lacked mechanical durability and flexibility, limiting its applicability in wearable devices subjected to continuous deformation. Despite such advances, most ionogels remain restricted to single‐mode sensing and often fail under structural or electrical failure under elevated temperatures. Dual‐functional materials capable of simultaneously detecting temperature and mechanical changes are still rare, particularly those based on sustainable resources. More critically, few ionogels retain functional stability after direct flame exposure, owing to irreversible network degradation and ion loss.

Herein, we fabricate a functional glycol lignin (Gly‐lignin) ionogel (GLI) that combines flame‐retardant, waterproof, and sensing capabilities, specifically designed to address the challenges associated with fire‐prone and harsh environments. Constructed from Gly‐lignin, 1‐butyl‐3‐vinylimidazolium tetrafluoroborate (BVIMBF_4_), and benzyl methacrylate (BZMA) within a 1‐butyl‐3‐methylimidazolium bis (trifluoromethylsulfonyl) imide (BMIMTFSI) system, the ionogel forms a durable 3D network via chemical and physical crosslinking, achieving a tensile strength of 5.8 MPa and adhesion of 30.4 kPa on flame‐retardant gloves. Notably, the ionogel exhibits significant flame‐retardant behavior by forming a protective carbonized/B_2_O_3_ layer and chemical barrier gases (contains CO_2_, HF, BF_3_) upon combustion, while retaining its temperature and motion sensing capabilities even after exposure to fire. This dual functionality positions it as a promising candidate for smart firefighting sensors that can enhance situational awareness and safety.

The successful integration of the sensor module into a real‐time monitoring system demonstrates its potential to provide reliable safety alerts and monitor conditions dynamically, offering a resilient and multifunctional solution suitable for deployment in extreme and hazardous environments. In addition, GLI exhibits excellent biocompatibility and environmental degradability, expanding its potential for use in sustainable and wearable electronic systems. This work represents a notable advancement in the development of ionogels by effectively combining fire resistance, environmental adaptability, and real‐time sensing capabilities, paving the way for novel applications in smart firefighting and wearable safety technologies.

## 2 Results and Discussion

### 2.1 Synthesis and Structural Characterization of GLI

GLI is designed by integrating flame‐retardant, waterproof, and sensing capabilities, making it well‐suited for use in harsh environments. The synthesis process is carried out in four stages: the preparation of Gly‐lignin and BVIMBF_4_, the dissolution of bio‐polymeric macromolecules and synthetic polymer monomers, followed by thermally induced copolymerization of BVIMBF_4_/BZMA as well as sol‐gel transformation of Gly‐lignin, culminating in the formation of an interpenetrating network.^[^ ^19^ ^]^ This composite network integrates the aromatic hydrocarbon structure of lignin, the conjugated electron cloud of imidazolium rings, and the positive‐negative ions in halogenated poly(ionic liquid) (PIL), and the lateral benzyl groups in PBZMA, thus exhibiting outstanding fire resistance, water repellency, electrical conductivity, and tensile strength.

**Figure 1:**
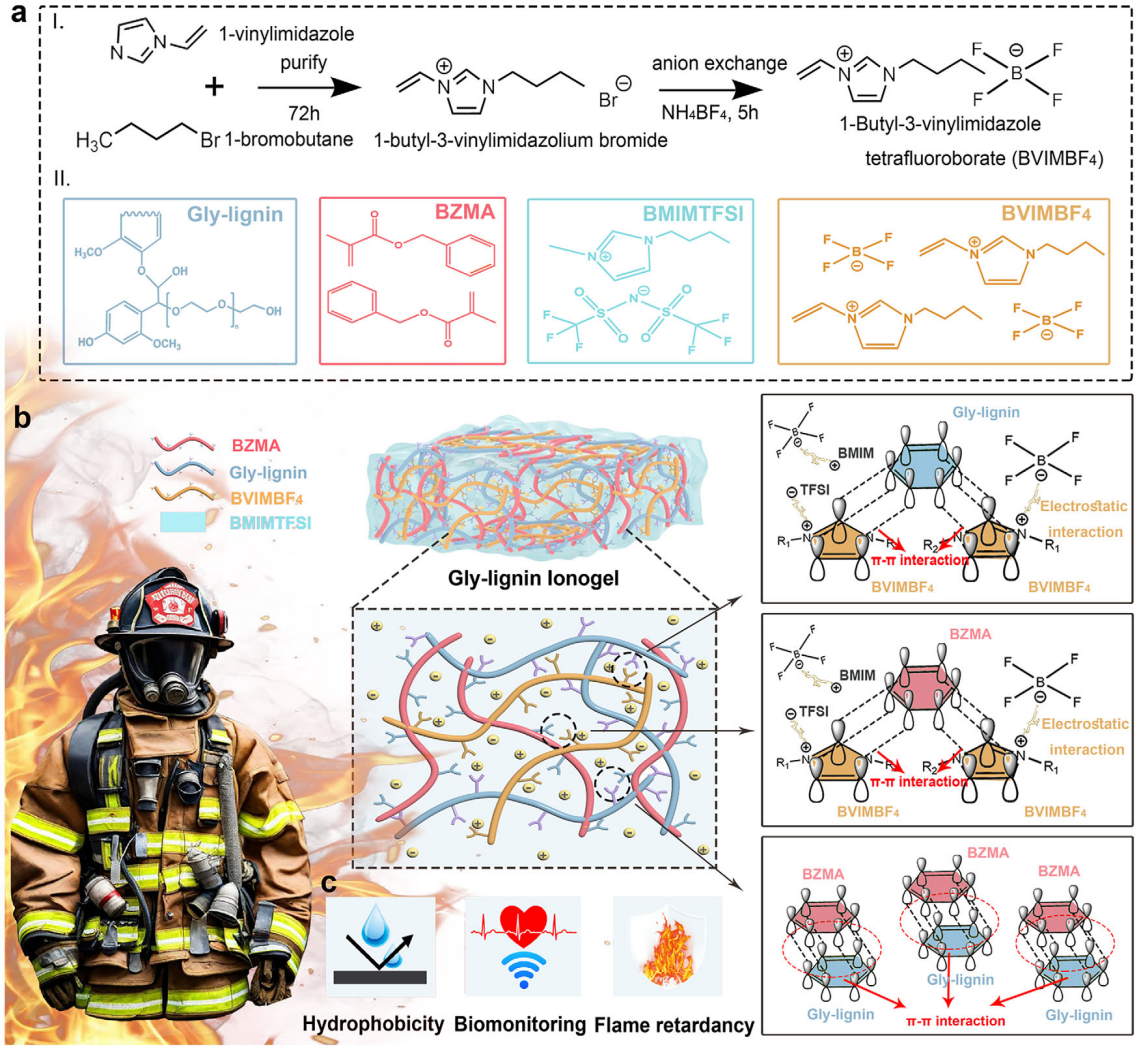
Schematic of preparation and application of GLI. a) Synthesis of Gly‐lignin and b) BVIMBF_4_. c) Structural formulas of BZMA, BMIMTFSI, and BVIMBF_4_. d) Schematic diagram of GLI structure and internal interactions.

To address the inherent drawbacks of lignin, such as its poor solubility in common organic solvents and high glass transition temperature,^[^ ^20^ ^]^ polyols are grafted onto lignin side chains through an etherification reaction during extraction (**Figure** **1**a), yielding Gly‐lignin with improved solubility and a lower melting point.^[^ ^21^ ^]^ The synthesis of BVIMBF_4_ begins with a nucleophilic substitution reaction^[^ ^22^ ^]^ between 1‐vinylimidazole and 1‐bromobutane, resulting in the formation of 1‐butyl‐3‐vinylimidazolium bromide (BVIMBr). An anion exchange^[^ ^23^ ^]^ between BVIMBr and NH_4_BF_4_ then follows to yield the desired flame‐retardant BVIMBF_4_ (Figure 1b). Ultimately, for ionogel formation, Gly‐lignin, BVIMBF_4_, and BZMA are uniformly dissolved in the BMIMTFSI medium, following thermal initiation of copolymerization and a sol‐gel transition, an ideal dual‐functional ionogel sensor, suitable for high‐temperature environments, is obtained.

Within this three‐component GIL (Figure 1c), multiple interactions including covalent bonding, *π*–*π* conjugation, hydrogen bonding,^[^ ^24^ ^]^ and electrostatic interactions, contribute to the overall robustness, stability, and energy dissipation capacity of the network.^[^ ^25^ ^]^ Notably, the addition of PBZMA significantly enhances the toughness and dissipation properties of the GIL, as it participates in three types of interactions (covalent bonding, *π*–*π* conjugation, and hydrogen bonding). *π*–*π* conjugation among the aromatic rings of Gly‐lignin, BZMA, and BVIMBF_4_ (Figure 1d) plays a critical role in reinforcing both intramolecular and intermolecular cohesion within the polymer matrix. Beyond structural stabilization, such interactions enable the reversible redistribution of mechanical energy through dynamic bond reorganization during deformation, contributing to enhanced toughness and energy dissipation without inducing significant irreversible energy loss.^[^ ^26^ ^]^ Additionally, the incorporation of ionic liquids PBVIMBF_4_ and BMIMTFSI introduces additional crosslinking sites through electrostatic interactions between their respective cations (BVIM⁺ and BMIM⁺) and anions (BF_4_⁻ and TFSI⁻). This dynamic ionic network enhances both mechanical stability and ionic conductivity, promoting efficient charge transport and maintaining robust conductive pathways within the ionogel matrix.^[^ ^27^ ^]^

### 2.2 Mechanical Properties of Ionogels

**Figure 2:**
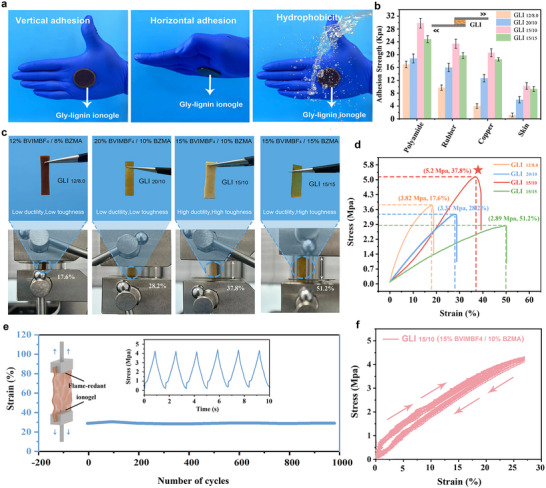
Mechanical properties of GLIs. a) Adhesion capabilities of GLIs at various environmental conditions. b) Adhesion strength of different formulations of GLIs on various substrates. c) Physical stretching test images of different ionogel formulations. d) Stress–strain curves of GLIs. e) Tensile cycling behavior of GLI_15/10_ over 1000 cycles. f) Stress–strain analysis of GLI_15/10_ during repeated mechanical deformation.

The mechanical properties of GLI are thoroughly characterized, focusing on its adhesive strength, tensile performance, and fatigue resistance. Under diverse environmental conditions, GLI exhibits remarkable adhesive performance and adaptability (**Figure** **2**a). The adhesive strength values of GLI on substrates such as polyamide, rubber, copper, and skin vary with the formulations (Figure 2b). Notably, GLI_15/10_, comprising 15% BVIMBF_4_ and 10% BZMA, demonstrates exceptional adhesion, achieving 30.4 kPa on polyamide, 23.5 kPa on rubber, 21.8 kPa on copper, and 11.1 kPa on skin. The strong interfacial bonding can be attributed to multiple molecular interactions. Hydrogen bonding facilitated by the hydroxyl‐rich Gly‐lignin enhances adhesion to polar substrates such as polyamide while *π*–*π* stacking interactions between the benzyl groups in BZMA and aromatic structures in rubber contribute to adhesion stability. Additionally, electrostatic interactions from the BVIMBF_4_ ionic liquid strengthen bonding with metal surfaces such as copper. To further examine the long‐term adhesion behavior of GLI, its stability on different substrates was monitored over time, revealing substrate‐dependent variations in adhesion retention (Figure S1, Supporting Information). Stress–strain curves reveal that GLI_15/10_ attains a maximum tensile strength of 5.8 MPa and an optimal elongation at a break of 37.8% (Figure 2c,d), again outperforming other formulations.

The exceptional performance of GLI_15/10_ originates from the synergistic interactions between PBVIMBF_4_ and BZMA. At 15 wt%, PBVIMBF_4_ forms a dense ionic crosslinked network, significantly enhancing tensile strength and adhesion while mitigating excessive rigidity.^[^ ^28^ ^]^ Meanwhile, 10 wt% BZMA provides the necessary flexibility to preserve the mechanical integrity of the matrix.^[^ ^29^ ^]^ In contrast, formulations with lower concentrations of PBVIMBF_4_ and BZMA (12 and 8 wt%) result in insufficient crosslinking, compromising mechanical properties, whereas higher concentrations (20 wt% PBVIMBF_4_ or 15 wt% BZMA) lead to either excessive rigidity or over‐softening of the matrix.

Fatigue resistance is assessed by subjecting the GLI_15/10_ to tensile cycling tests. After 1000 stretching cycles, the ionogel consistently maintains ≈38% strain (Figure 2e), demonstrating excellent fatigue resistance and mechanical stability under repeated stress. The multiple physical crosslinked network effectively resists mechanical fatigue through ionic‐dipole interactions,^[^ ^30^ ^]^ maintaining structural integrity—an essential property for flexible and wearable applications. Cyclic tensile testing of GLI_15/10_ (Figure 2f) shows narrow and highly reproducible hysteresis loops across repeated loading–unloading cycles. The limited hysteresis area indicates that energy dissipation during deformation primarily originates from reversible bond reorganization rather than irreversible frictional loss.^[^ ^31^ ^]^ This reversible dynamic mechanism accounts for the observed low internal friction and contributes to excellent elastic recovery. In addition, the nearly linear stress–strain trajectories during both loading and unloading confirm stable elastic behavior, underscoring the mechanical resilience of the ionogel network under cyclic strain.

### 2.3 Flame‐Retardant Properties of GLI

**Figure 3:**
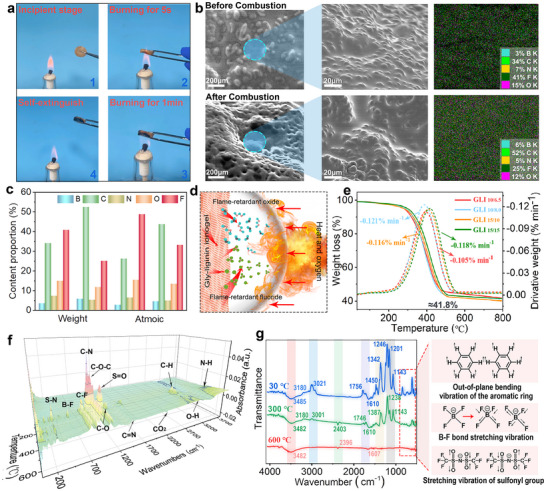
Thermal properties of GLI. a) Stages of combustion of GLI. b) SEM images before and after combustion. c) Elemental analysis of the ionogel via EDS. d) Schematic representation of the flame‐retardant mechanism. e) Thermogravimetric analysis of GLIs with different proportions. f) 3D TG‐FTIR spectra depicting the evolution of key functional groups. g) FTIR spectra at various temperatures.

Flame‐retardant properties of GLI under combustion conditions are thoroughly examined. During combustion tests using an alcohol burner, the ionogel undergoes several stages: initial ignition, burning for 5s, burning for 1 min, and self‐extinguishing after flame exposure. No significant changes were observed during the initial ignition and after 5s of burning, confirming the ionogel's inherent stability against fire. After 1 min of exposure, a carbonized layer forms in the flame‐contacted region, along with the formation of small solid particles (**Figure** **3**a). The non‐contacted area remains largely intact. Upon removal from the flame, the ionogel self‐extinguishes, further demonstrating its excellent flame‐retardant behavior^[^ ^32^ ^]^ and emphasizing its suitability for fire safety applications.

To further explore morphological and elemental changes before and after combustion, scanning electron microscopy (SEM) and energy‐dispersive X‐ray spectroscopy (EDS) were employed. SEM images of the ionogel show a dense and compact surface uniformly distributed with ≈50 µm particles before combustion (Figure 3b), which might be caused by the phase separation of Gly‐lignin from poly(BVIMBF_4_‐co‐BZMA) during the sol‐gel process. After combustion, the surface becomes porous and rougher, likely due to the thermal degradation of GIL and the formation and volatilization of obstructive compounds (including CO_2_ and a small quantity of HF and BF_3_,) from PBVIMBF_4_ and BMIMTFSI.

Elemental mapping and EDS analysis reveal a decrease in fluorine and nitrogen content after combustion, while carbon and boron levels increase (Figure 3c). These component changes confirm the formation of a carbonized layer and the emergence of flame‐retardant fluorine‐containing, boron‐containing, and CO_2_ flame‐retardant oxides during combustion (Figure 3d). Specifically, CO_2_ serves as a diluent, reducing oxygen availability,^[^ ^33^ ^]^ while B_2_O_3_ forms a protective barrier on the material's surface, which not only prevents further thermal degradation^[^ ^34^ ^]^ but also insulates the underlying material, reducing heat transfer and limiting oxygen diffusion. In the gas phase, HF and BF_3_ function as flame inhibitors by capturing highly reactive radicals that sustain combustion. HF reacts with hydroxyl radicals (•OH), forming water and releasing fluorine radicals (•F), which further scavenge hydrogen radicals (•H), thereby disrupting the radical chain reaction necessary for the flame.

Similarly, BF_3_ interacts with •OH and •H radicals, further reducing the concentration of these reactive species in the flame zone and preventing the continuation of the combustion process.^[^ ^35^ ^]^ The synergistic action of HF and BF₃ in radical quenching, combined with the formation of the B_2_O_3_ protective layer, significantly enhances the flame‐retardant performance of the ionogel. GLI_15/10_ exhibits a high char residue and low weight loss during TGA testing (Figure 3e), indicating its ability to maintain structural integrity and resist thermal decomposition, thereby demonstrating excellent thermal stability. Further insights into elemental and functional group transformations are provided by 3D TG‐FTIR spectra and FTIR analysis (Figure 3f,g). The volatilization of fluorine‐containing compounds is reflected in the declining B─F and C─F peaks,^[^ ^36^ ^]^ while the degradation of sulfonyl groups is indicated by reduced S‐N peaks (Figure 3f).^[^ ^36b^ ^]^ At 30 °C, FTIR spectra indicate an intact ionogel structure with distinct B─F bonds, aromatic rings,^[^ ^37^ ^]^ and sulfonyl groups.^[^ ^38^ ^]^ Upon heating to 300 °C, the decline in B─F and C─F peaks indicates the volatilization of fluorine‐based flame retardants such as BF_3_ and HF. At 600 °C, the FTIR spectrum reveals near‐complete decomposition of organic functional groups, including B─F and C─F bonds, suggesting the continued volatilization of these fluorine‐based retardants and a significant reduction in the iongoel's organic fraction. To quantitatively assess the fire safety performance of GLI_15/10_, cone calorimetry is performed under standardized combustion conditions (Figure S2, Supporting Information). GLI_15/10_ exhibits a prolonged time to ignition (51 s), a low peak heat release rate (165 kW m^−2^), and limited total heat release (25 MJ m^−2^). The effective heat of combustion (15 MJ kg^−1^) also supports incomplete combustion, aligning with the observed condensed‐phase barrier and gas‐phase flame inhibition. These results reinforce the intrinsic flame‐retardant behavior of the ionogel under standardized fire exposure.

Simultaneously, the formation of B₂O₃ is evident, marked by characteristic peaks^[^ ^39^ ^]^ at 1370 cm^−1^. This protective boron oxide layer enhances flame retardancy by blocking further degradation. Additionally, CO_2_ peaks^[^ ^40^ ^]^ at 2350 cm^−1^ are observed, confirming the release of CO_2_ during thermal degradation, which acts as a flame‐suppressing agent by diluting oxygen and limiting combustion. Furthermore, differential scanning calorimetry (DSC) is employed to further investigate the thermal behavior of GLI (Figure S3, Supporting Information). The results indicate a glass transition temperature (Tg) of 165.6 °C, demonstrating excellent thermal stability under elevated temperatures. Additionally, the observed freezing point of ≈−52 °C suggests the material's suitability for low‐temperature environments. The broad operating temperature range underscores its adaptability to extreme thermal conditions, further supporting its potential for applications requiring thermal resilience.

### 2.4 Thermal and Mechanical Response of Ionogels

**Figure 4:**
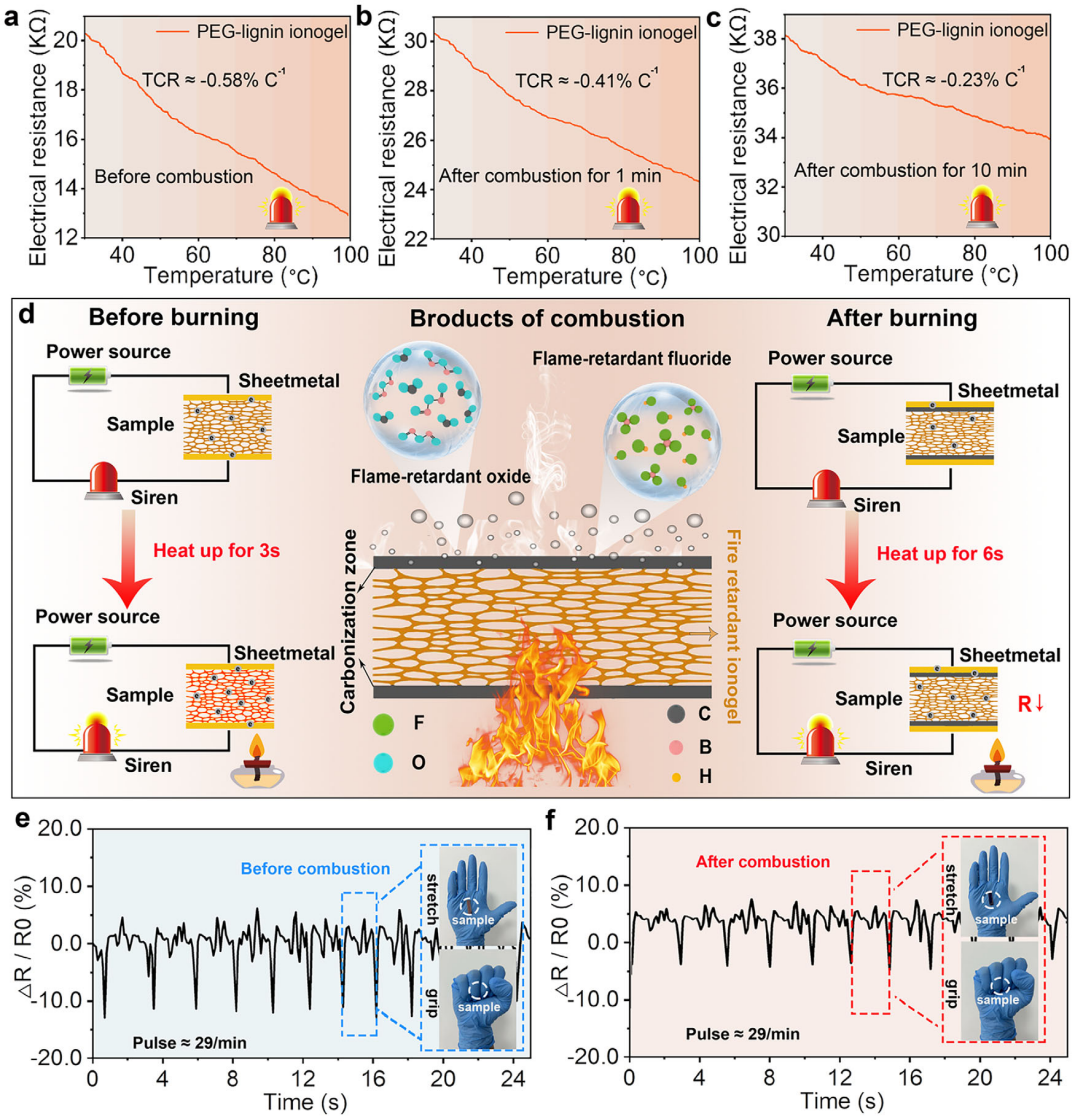
Thermal and mechanical response of GLI_15/10_. a) Electrical resistance‐temperature relationship of GLI_15/10_ before burning, b) burning for 1 min, and c) burning for 10 min. d) Schematic illustrating the effect of carbonized layer formation on temperature sensitivity. e) Strain response under cyclic deformation of GLI_15/10_ before and f) after combustion.

The thermal and mechanical sensing response of GLI_15/10_ is comprehensively analyzed in **Figure** **4**. In terms of thermal sensing response, GLI_15/10_ exhibits negative temperature coefficient (NTC) behavior, where resistance decreases with rising temperature.^[^ ^41^ ^]^ This behavior is attributed to enhanced charge carrier mobility at higher temperatures, which reduces ion transport resistance in the gel matrix and improves conductivity.^[^ ^23^, ^42^ ^]^ Additionally, hopping conduction and tunneling effects are strengthened within particles and between adjacent nanosheets, allowing charge carriers to overcome local barriers more easily, ultimately leading to improved overall conductivity and decreased resistance. The sensitivity of GLI_15/10_ is defined by the temperature coefficient of resistance (TCR),^[^ ^43^ ^]^ which is calculated using Equation (1):

(1) $$\mathrm{TCR}=\frac{R-R_{0}}{R_{0}}\times \frac{1}{\Delta T}$$

where *R* and *R_0_* are the resistance at the measured temperature and initial temperature (40 °C) of the sensor and *∆T* is the change in applied temperature. The calculated TCR value of the ionogel is −0.58% °C^−1^ (Figure 4a), indicating its potential for development as a temperature sensor. To evaluate thermal stability under fluctuating conditions, multiple temperature cycling tests (Figure S4, Supporting Information) were performed. The resistance response remained consistent throughout, indicating that the dynamic ionic and supramolecular network maintains stable conductive pathways during thermal variations.^[^ ^44^, ^45^ ^]^ Notably, GLI_15/10_ undergoes progressive carbonization from the exterior to the interior during intense combustion (Figure S5, Supporting Information), effectively preserving the internal ionic conduction network.

It is worth noting that a GLI_15/10_ ionogel membrane with a thickness of 3 mm maintains excellent adhesion, electrical conductivity, and temperature sensing performance (Figure 4b,c; Figure S6, Supporting Information) until it is fully consumed after 15 min of sustained flame exposure at ≈1000 °C. It continuously provides monitoring and early warning functionality as long as the resistance signal exceeds the defined threshold. The observed performance degradation at a certain degree is mainly attributed to structural evolution, i.e., the thermal decomposition of the PIL gel framework along with the lignin network to form a carbonized outer layer, which hinders ion transport and electrical conductivity.^[^ ^46^ ^]^ Moreover, the membrane thickness can be adjusted as needed to extend its operational lifespan in fire scenarios.

Before combustion, the ionogel exhibits a uniform structure with high ionic conductivity, enabling rapid detection of temperature fluctuations and timely activation of the alarm system (response time of 3 s). After combustion, a carbonized layer forms on the surface, acting as a barrier that reduces ion mobility^[^ ^47^ ^]^ and increases response time. Simultaneously, degradation of the internal gel network, including the breakdown of Gly‐lignin, BVIMBF_4_, and BZMA components, further disrupts ion conduction by compromising the polymer matrix and ion transport pathways. The combination of surface carbonization and internal degradation leads to a significant reduction in ionic conductivity,^[^ ^48^ ^]^ which slows the thermal response (response time of 6 s). However, GLI_15/10_ retained its temperature sensing capability post‐combustion, demonstrating its potential as a temperature alarm in harsh environments (Figure 4d).

The strain response of our GLI_15/10_ under cyclic mechanical deformation is shown in Figure 4e. Before combustion, the resistance (ΔR/R_0_) of GLI_15/10_ exhibits stable and repeatable signal responses to stretching and gripping motions, with a response speed of ≈29 cycles per minute. After combustion, both response speed and amplitude decrease slightly, yet the material retains significant stability and repeatability (Figure 4f). This long‐lasting mechanosensing performance, together with the thermosensing capability demonstrated above, underscores the dual‐sensing potential of GLI_15/10_ for continuous monitoring of mechanical deformation and environmental temperature in fire scenarios.

### 2.5 Dual Sensing Application

**Figure 5:**
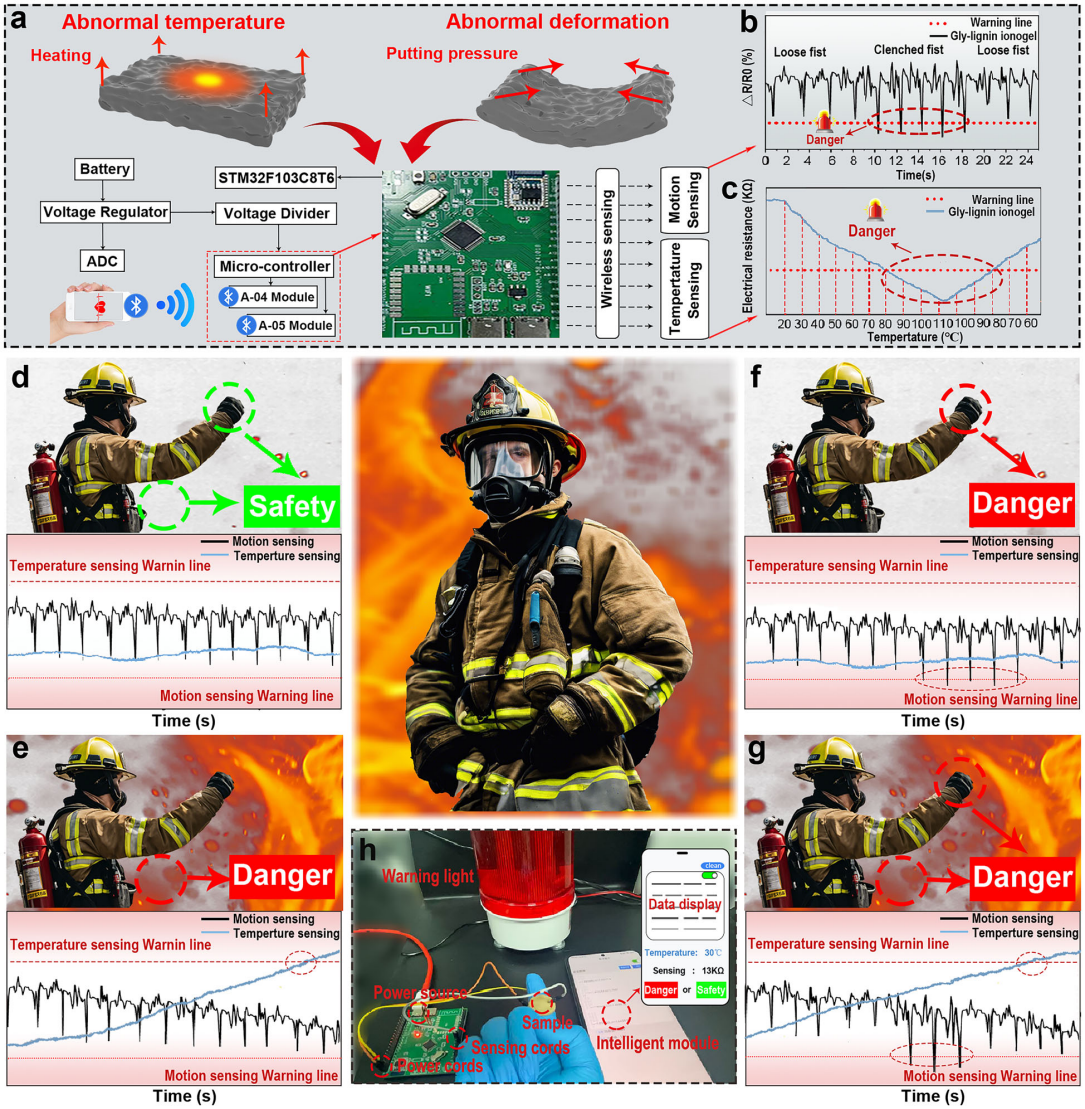
Dual sensing application of GLI_15/10_. a) Schematic of GLI_15/10_‐based sensor module. b) Motion sensing application of GLI_15/10_ sensor module. c) Temperature sensing application. d) Scenario under safe conditions with corresponding data display. e) Elevated temperature triggering danger alert with corresponding data display. f) Abnormal motion triggering danger alert with corresponding data display. g) Simultaneous abnormal motion and elevated temperature triggering danger alert with corresponding data display. h) Experimental setup for dual sensing capability.

The operational performance of the GLI_15/10_ sensor module integrates temperature and deformation sensing for real‐time monitoring. The system includes a power supply battery, a voltage regulator, an STM32F103C8T6 microcontroller for data acquisition and control, and a voltage divider for signal conditioning (**Figure** **5**a). When abnormal temperature or deformation events are detected, the microcontroller activates an alarm system and enables wireless data transmission via Bluetooth communication, thereby achieving remote monitoring functionality. The motion sensing module operates by detecting resistance changes (*ΔR/R0*) caused by hand gestures transitioning between clenched and loose states. Temperature sensing is validated through a decrease in resistance as temperature rises, triggering an alarm upon exceeding a predefined threshold (Figure 5b,c). These findings underscore the ionogel's suitability for overheating detection and abnormal motion monitoring, making it highly suitable for fire safety and temperature monitoring applications.^[^ ^49^ ^]^

The sensing and monitoring performance of the GLI_15/10_ sensor module under simulated fire conditions highlights its effectiveness. When both environmental temperature and hand gestures remain within normal ranges, the system classifies the scenario as‚ “Safety” as indicated by the corresponding mechanical and thermal sensing data within the predefined threshold (Figure 5d). In this state, the data confirms stable conditions and a safe environment, with no alarm triggered. Conversely, when either the temperature, hand gesture, or both deviate from the normal range, the system classifies the situation as “Danger” and triggers an alert.

Specifically, when the hand gesture remains within the normal range, an increase in ambient temperature beyond the predefined threshold triggers the alarm (Figure 5e). Similarly, an abnormal hand gesture, deviating from the normal range while the ambient temperature remains stable, activates the alarm (Figure 5d). Furthermore, when both temperature and hand gesture signals exceed the established safety limits, the alarm is triggered (Figure 5f), highlighting the module's ability to detect multiple concurrent threats. The experimental setup validating the performance of the sensor module is shown in Figure 5h. The system integrates the GLI_15/10_ sensor with a development board and power supply, connecting to a mobile application for real‐time data visualization and monitoring. This configuration enables effective detection of anomalies and timely alert triggering, as can be seen in Movie S1 (Supporting Information). The experimental results confirm the dual sensing capability of the module (temperature and deformation), underscoring its practical potential to enhance situational awareness and safety in fire‐related environments.

### 2.6 Multifunctional Evaluation of GLI_15/10_

**Figure 6:**
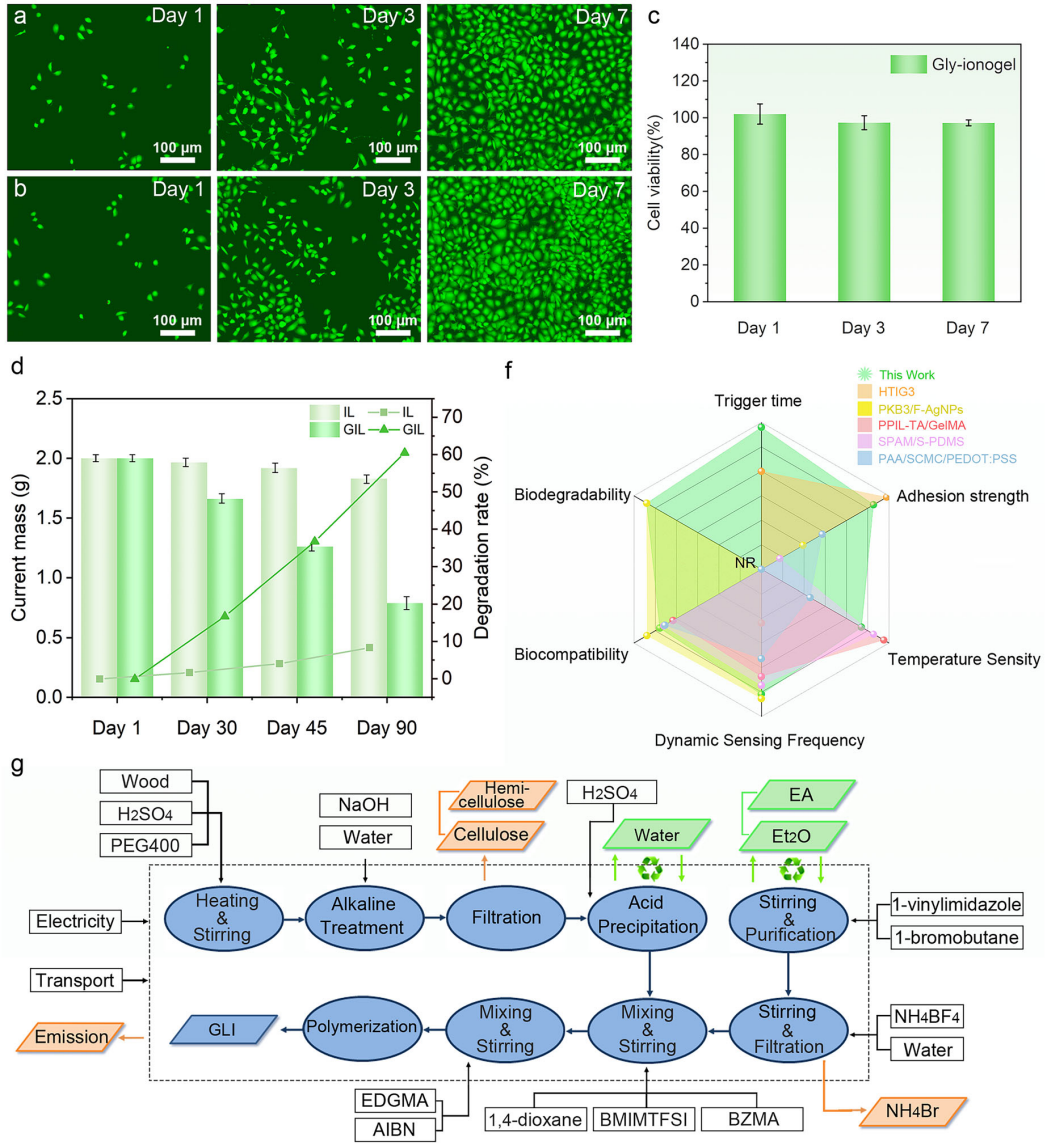
Biological evaluation and performance comparison of GLI_15/10_. a) Fluorescence microscopy images of cells under standard conditions for 1, 3, and 7 days. b) Fluorescence microscopy images of cells cultured on GLI_15/10_ for 1, 3, and 7 days. c) Cell viability analysis of vascular endothelial cells on different substrates. d) Remaining mass and degradation rate of GLI_15/10_ and LI over time. e) System boundary diagram of the GLI_15/10_ production process. f) Performance comparison of GLI_15/10_ with reported gel‐based materials.

Biocompatibility is a critical parameter for evaluating the potential of ionogels in wearable biomedical and environmental applications.^[^ ^50^ ^]^ Here, the cytocompatibility of GLI_15/10_ was systematically assessed using human umbilical vein endothelial cells (HUVECs). HUVECs cultured under standard conditions exhibited healthy morphology and steadily increasing cell density from Day 1 to Day 7 (**Figure** **6**a). Likewise, cells co‐cultured with GLI_15/10_ samples (1 cm × 1 cm) displayed comparable proliferation behavior, as demonstrated by the live/dead fluorescence staining images (Figure 6b). The predominance of strong green fluorescence (calcein‐AM) indicated high viability and minimal cytotoxicity. In parallel, the CCK‐8 assay (Figure 6c) revealed that the cell viability on GLI_15/10_ remained above 98% throughout the culture period, comparable to the control group. These results collectively confirm the excellent biocompatibility of GLI_15/10_.

Beyond biocompatibility, environmental degradability is pivotal for the development of sustainable and electronic materials. The degradability of GLI_15/10_ was investigated under natural outdoor conditions over a 90‐day period, with gly‐lignin‐free ionogels (LI) used as a reference. As depicted in Figure S7 (Supporting Information), GLI_15/10_ exhibited progressive structural disintegration, beginning with surface erosion by Day 15 and culminating in pronounced material disintegration by Day 90. In contrast, LI samples retained structural integrity throughout, indicating poor environmental responsiveness. Residual mass analysis (Figure 6d) further substantiated these observations, revealing a degradation rate exceeding 60% for GLI_15/10_, while LI experienced only ∼8% mass loss under identical conditions. This enhanced degradability is attributed to gly‐lignin incorporation, which promotes hydrolytic and microbial breakdown under ambient environments.^[^ ^51^ ^]^ These findings underscore the environmentally responsive and transient nature of GLI_15/10_, advancing its potential for biodegradable or disposable electronics in ecologically sensitive contexts.

In order to provide a holistic view of material sustainability, a cradle‐to‐gate life cycle assessment (LCA) was conducted to systematically quantify the environmental impacts associated with GLI_15/10_ production.^[^ ^52^, ^53^ ^]^ The system boundary diagram (Figure 6e) encompasses all upstream and midstream processes involved in its fabrication, including the preparation of bio‐based and synthetic precursors (Gly‐lignin, PEG400, BVIMBF_4_, BZMA, BMIMTFSI). A full inventory of input and output flows is provided in Table S1 (Supporting Information). The assessment covered eleven midpoint impact categories: abiotic depletion of elements (ADP‐elements) and fossil fuels (ADP‐fossil), global warming potential (GWP100a), ozone depletion potential (ODP), acidification potential (AP), eutrophication potential (EP), photochemical oxidant creation potential (POCP), human toxicity (HTP), freshwater aquatic ecotoxicity (FATEP), marine aquatic ecotoxicity (MATEP), and terrestrial ecotoxicity (TEP).

As shown in Figure S8 (Supporting Information), contribution analysis revealed that BMIMTFSI and BZMA were the predominant environmental hotspots, together accounting for over 60% of the burdens in categories such as GWP, ADP‐fossil, and POCP. Secondary contributors included NH_4_BF_4_ and PEG400, particularly contributing to ODP and toxicity‐related categories. In contrast, auxiliary inputs such as electricity, water, and transportation contributed less than 5% across all categories. To benchmark the environmental performance of GLI_15/10_, a comparative analysis was conducted against a previously reported green ionogel (S‐PAM/S‐PDMS) developed for health‐monitoring applications (Figure S9 and Tables S2 and S3, Supporting Information). GLI_15/10_ exhibited a similarly low environmental burden across key indicators, particularly in GWP, EP, and ADP‐fossil, highlighting its potential as a sustainable and high‐performance material platform for next‐generation bioelectronic and fire‐resilient applications.

To further evaluate its multifunctionality, GLI_15/10_ was compared with representative ionogel and hydrogel systems^[^ ^54^, ^55^, ^56^, ^57^, ^58^ ^]^ (Figure 6f). Benchmark metrics included tensile strength, ionic conductivity, adhesion, flame retardancy, and degradability. Unlike conventional gel systems that typically exhibit trade‐offs among mechanical strength, environmental adaptability, and sensing capabilities, GLI_15/10_ integrates all these features within a single material matrix. It combines high tensile strength and adhesion with excellent ionic conductivity, while simultaneously demonstrating outstanding flame retardancy, biocompatibility, and environmental degradability. This well‐balanced performance profile establishes GLI_15/10_ as a versatile and high‐performance platform for next‐generation wearable, fire‐resilient, and biodegradable electronics in complex or ecologically sensitive environments.

## 3 Conclusion

In this work, a multifunctional GLI with flame‐retardant, waterproof, and sensing capabilities is developed. Synthesized from Gly‐lignin, BVIMBF_4_, and BZMA within the BMIMTFSI system, the ionogel forms a stable 3D interpenetrating network through both chemical and physical crosslinking. This structure endows GLI_15/10_ with excellent tensile strength (5.8 MPa) and strong adhesion (30.4 kPa on flame‐retardant gloves). The ionogel demonstrates significant flame‐retardant behavior (weight loss of 41.8% at 800 °C), forming a protective carbonized/B_2_O_3_ layer and chemical barrier gases upon combustion. Moreover, GLI_15/10_ possesses both temperature and motion‐sensing capabilities even after exposure to fire, underscoring its potential as a dual‐functional sensor for fire safety. Integrated into a real‐time monitoring system, the sensor module enhances situational awareness and safety in hazardous environments. Notably, GLI_15/10_ also demonstrates excellent biocompatibility and environmental degradability, further broadening its potential toward sustainable and wearable electronic applications. In short, this work presents a novel multifunctional ionogel that combines mechanical robustness, fire resistance, as well as environmental sensing, and real‐time monitoring capacities, positioning it as a promising candidate for rescue operations in fire‐prone environments and other hazardous conditions.

## 4 Experimental Section

### Materials and Chemicals

Poplar sawdust (100 mesh) was purchased from Tianlai Wood Co., Ltd. (Shandong, China). Concentrated sulfuric acid was obtained from Yonghua Chemical Co., Ltd. (Shanghai, China), and diethyl ether was purchased from Xilong Scientific Co., Ltd. (Guangdong, China). NaOH, polyethylene glycol 400 (PEG400), benzyl methacrylate (BZMA), 1‐butyl‐3‐methylimidazolium bis(trifluoromethylsulfonyl)imide (BMIMTFSI), ethylene glycol dimethacrylate (EGDMA), azobisisobutyronitrile (AIBN), ethyl acetate, 1‐butyl‐3‐vinylimidazolium bromide (BVIMBr), and ammonium tetrafluoroborate (NH₄BF₄) were all purchased from Aladdin (Shanghai, China) and used as received.

### Modification of Lignin

Gly‐lignin was obtained by mixing dried poplar sawdust with PEG 400 at a ratio of 1:5, followed by the addition of 0.04%wt concentrated sulfuric acid. The mixture was stirred at 140 °C for 1.5 h, then cooled to room temperature before adding a 0.02%wt aqueous solution of NaOH and stirring for an additional 2 h, resulting in a brownish mixture. Solid–liquid separation was carried out using a Buchner funnel and a circulating water vacuum pump. The obtained liquid was treated with a certain amount of sulfuric acid until the solution reached a pH of 1, followed by the addition of a specific volume of deionized water. After settling, the precipitate was dried, ground, and characterized to yield the modified polyethylene glycol lignin.

### Modification of BVIMBF_4_

Taking 0.11 mol of 1‐vinylimidazole, it was drip‐wise added to a solution containing 0.1 mol of 1‐bromobutane in a round‐bottom flask, and the resulting mixture was stirred for 72 h. The mixture was subsequently washed three times with diethyl ether and ethyl acetate to remove the upper layer of the resulting biphasic solution, yielding the crude product in the lower layer, which was then dried in an oven for 12 h to obtain colorless and transparent BVIM‐Br (1‐butyl‐3‐vinylimidazolium bromide). An appropriate amount of BVIM‐Br and NH_4_BF_4_ (3.47 and 2.77 g, respectively) were dissolved in water with vigorous stirring for 5 h. Upon allowing the mixture to stand, a white precipitate formed, which was subsequently collected by filtration and subjected to low‐temperature drying to yield BvimBF_4_ (1‐Butyl‐3‐vinylimidazole tetrafluoroborate)_._

### Modification of Gly‐Lignin Ionogel

Dissolve Gly‐lignin in 1,4‐dioxane and stir for 6 h to achieve complete dissolution. Add a certain proportion of BVIMBF_4_ and BZMA to the solution. To enhance the conductivity of the gel, add an appropriate amount of BMIMTFSI to the mixture, followed by the addition of a cross‐linker (EGDMA) and an initiator (AIBN). Continue stirring for 10 min. Subsequently, place the solution in a vacuum oven for polymerization under vacuum conditions at 80 °C for 8 h, ultimately yielding a Gly‐lignin‐based ionogel.

### Adhesion and Tensile Properties

The adhesion performance of the ionogel was evaluated using a lap shear method. The ionogel (4 cm × 2 cm × 1 cm) was placed between two substrates. A mechanical testing machine (CMT6104, Mts Systems Co., Ltd.) was used to stretch the substrates at a speed of 50 mm⋅min^−1^ at room temperature. The shear strength was determined as the maximum force applied per unit area. The mechanical properties of the ionogel were evaluated by clamping the GLIs (4 cm × 2 cm × 1 cm) with fixtures and performing uniaxial tensile testing at a constant strain rate of 100 mm min^−1^ until fracture. The stress–strain curve was obtained by calculation.

### Thermal Stability

Thermogravimetric (TG) analysis was used to evaluate the thermal stability of GLIs (TG209F1, NETZSCH, Germany). The GLIs were cut into small pieces, with each piece weighing ≈5 mg, and placed in an aluminum crucible, with an empty crucible used as a reference. Under a nitrogen atmosphere, the temperature was increased from room temperature to 800 °C at a heating rate of 20 °C min^−1^. The residual char content at 800 °C was evaluated, and derivative thermogravimetric (DTG) data were collected. To investigate the structural evolution of GLI15/10 during thermal decomposition, thermogravimetric analysis coupled with Fourier transform infrared spectroscopy (TG‐FTIR, TGA2 by Mettler Toledo and Nicolet iS50 by Thermo Fisher, Switzerland) was employed in the temperature range of 30–600 °C. In addition, the microstructure of GLI15/10 before and after combustion was further examined using scanning electron microscopy (SEM, SU8010, Hitachi, Japan).

### Cytocompatibility

The cytocompatibility of GLI was investigated by human umbilical vascular endothelial cells. HUVECs (4103 cells/well) were inoculated into 96‐well plates and cultivated for 12 h. Then the HUVECs were co‐cultured with the sample (1cm×1cm). After co‐culturing for 1,3 and 7 days, CCK‐8 solution (10% in DMEM, 100 µL) was added into each well to incubate for another 1 h. Eventually, the absorbance of suspensions was recorded at 450 nm by a microplate reader (Thermo, Multiskn Go, USA). Besides, live/dead assays were stained with calcein‐AM/propidium iodide and observed by an inverted fluorescence microscope (Nikon ECLIPSE Ti).

### Environmental Sustainability

The aim of this study was to quantify the environmental impact of the GLI_15/10_. The functional unit was defined as the production of 1 ton of GLI_15/10_, with mass and energy flow data sourced from information retrieval and process simulation (Table S1, Supporting Information). Life cycle assessment (LCA) was conducted using a “cradle‐to‐gate” approach, covering raw material preparation, the film production process, and the recycling of reusable resources, while considering the entire life cycle of all chemicals used in the experiment. LCA was conducted using SimaPro_9.5 software (https://support.simapro.com/). In the life cycle impact assessment (LCIA), the CML‐IA methodology was employed, and eleven environmental impact categories were evaluated (Table S2, Supporting Information). For further details, please refer to the Supporting Information.

### Thermal Stability

Thermogravimetric (TG) analysis was used to evaluate the thermal stability of GLIs (TG209F1, NETZSCH, Germany). The GLIs were cut into small pieces, with each piece weighing ≈5 mg, and placed in an aluminum crucible, with an empty crucible used as a reference. Under a nitrogen atmosphere, the temperature was increased from room temperature to 800 °C at a heating rate of 20 °C min^−1^. The residual char content at 800 °C was evaluated, and derivative thermogravimetric (DTG) data were collected.

### Gly‐Lignin Thermoelectric Resistance Measurement

The temperature coefficient of resistance (TCR) of Gly‐lignin was determined from the linear relationship between resistance (R) and temperature difference (ΔT). The resistance of the ionogel was measured using a DC resistance meter (HC2515, Huace, China), while ΔT was measured using a thermodetector (SSN61, Yowexa, China).

### Fire Alarm Dual Sensor Test

The fire safety module consisted of a wireless signal alarm (LTE‐1101J, Yueqing, Dahui Electric Co., Ltd., China), an STM32 development board (F103C8T6, Youxin Electronic Co., Ltd., China), and the GLI_15/10_ (2 cm × 1 cm × 1 cm). During testing, real‐time resistance measurements were recorded via the A4 and A5 ports on the development board, capturing changes in finger bending and surrounding temperature. The recorded data were transmitted wirelessly to a Bluetooth device. If the resistance at either the A4 or A5 port exceeded the preset threshold, a signal was sent to activate the alarm device.

## Conflict of Interest

The authors declare no conflict of interest.

## Author Contributions

Z.Y. and H.Y. contributed equally to this work. Z.Y. did conceptualization, validation, investigation, visualization, software, wrote the original draft. H.Y. did validation, methodology, software. H.X. did investigation, software, validation. W.Z. did software, validation. S.S. did investigation, software. C.L. did investigation, validation. Y.W. did investigation, validation. J.L. did investigation. Q.S. did conceptualization, resources, reviewed & editing, supervision. D.Z. reviewed & editing, supervision. X.S. did conceptualization, resources, reviewed &editing, supervision.

## Supporting information



Supporting Information

Supplemental Movie 1

## Data Availability

The data that support the findings of this study are available from the corresponding author upon reasonable request.
