# Supplementary material for: Flame‐Retardant Ionogel Enabled by Lignin Molecular Networks for Fire Rescue
Source: Adv Sci (Weinh). 2025 Jun 25;12(35):e06901. doi: 10.1002/advs.202506901 (PMC12463069; doi:10.1002/advs.202506901)
Supplement: Supplementary file 1 — Supporting Information [file ADVS-12-e06901-s001.docx]

**SUPPORTING INFORMATION**

Flame-Retardant Ionogel Enabled by Lignin Molecular Networks for Fire Rescue

Zewei Ye^1,#^, Haomeng Yu^1,#^, Hongxia Xie^1^, Wenwen Zhu^1^, Shitao Shi^1^, Chencong Liu^1^, Yuanyuan Wang^1^, Jiaqi Liao^1^, Qingfeng Sun^1,^*, Dawei Zhao^2,^*, Xiaoping Shen^1,^*

1. College of Chemistry and Materials Engineering, Zhejiang A&F University, Hangzhou 311300, P. R. China
2. Key Laboratory on Resources Chemicals and Materials of Ministry of Education, Shenyang University of Chemical Technology, Shenyang 110142, P. R. China

^#^ Z. Ye and H. Yu contribute equally.

* Corresponding to: [xpshen@zafu.edu.cn](mailto:xpshen@zafu.edu.cn); [daweizhao@syuct.edu.cn](mailto:daweizhao@syuct.edu.cn); [qfsun@zafu.edu.cn](mailto:qfsun@zafu.edu.cn);

**Keywords:** Flame-retardant ionogel, Wearable sensors, Harsh Environments, Real-time monitoring

**I. Supplementary Figures**


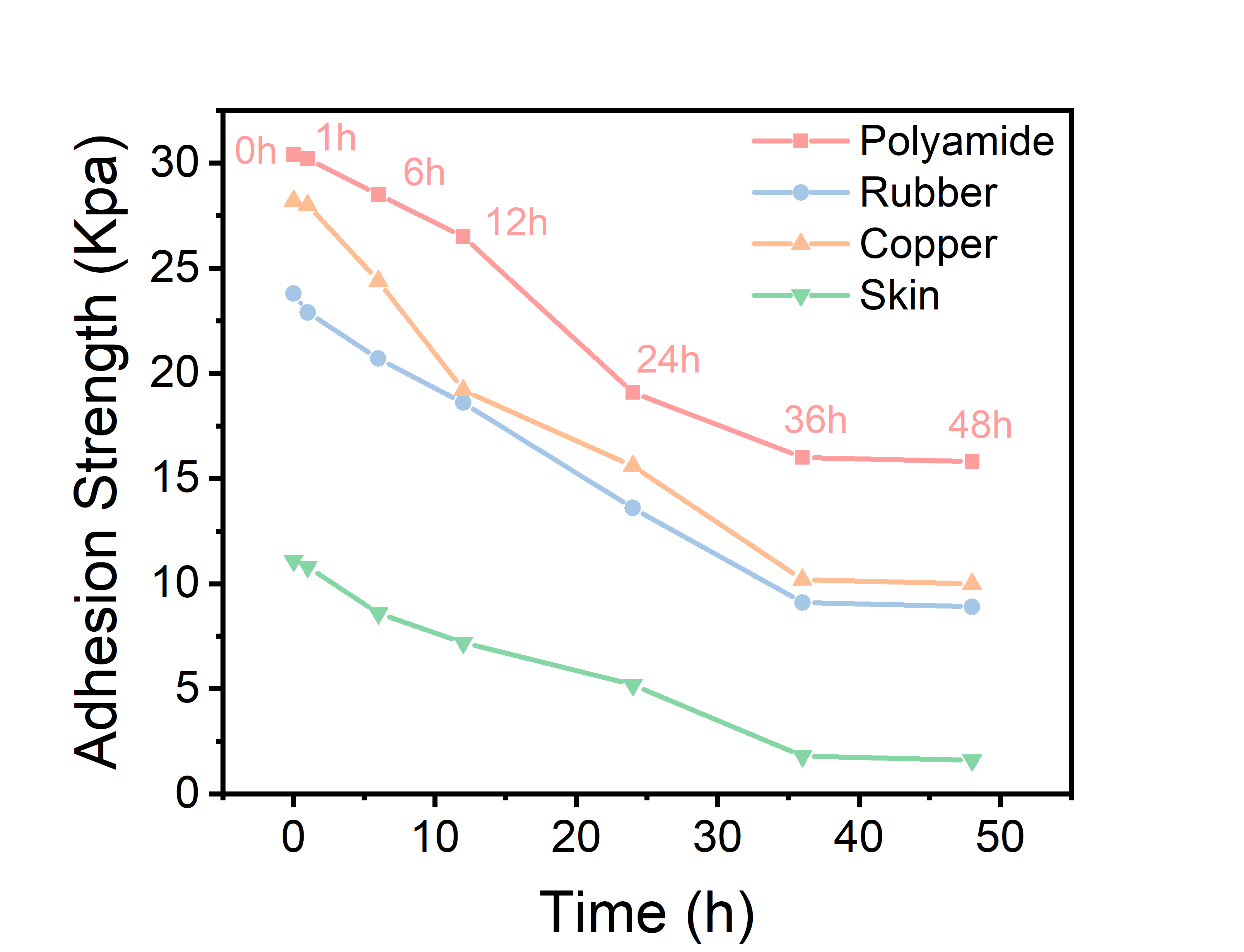


Figure S1: Adhesion strength variation.


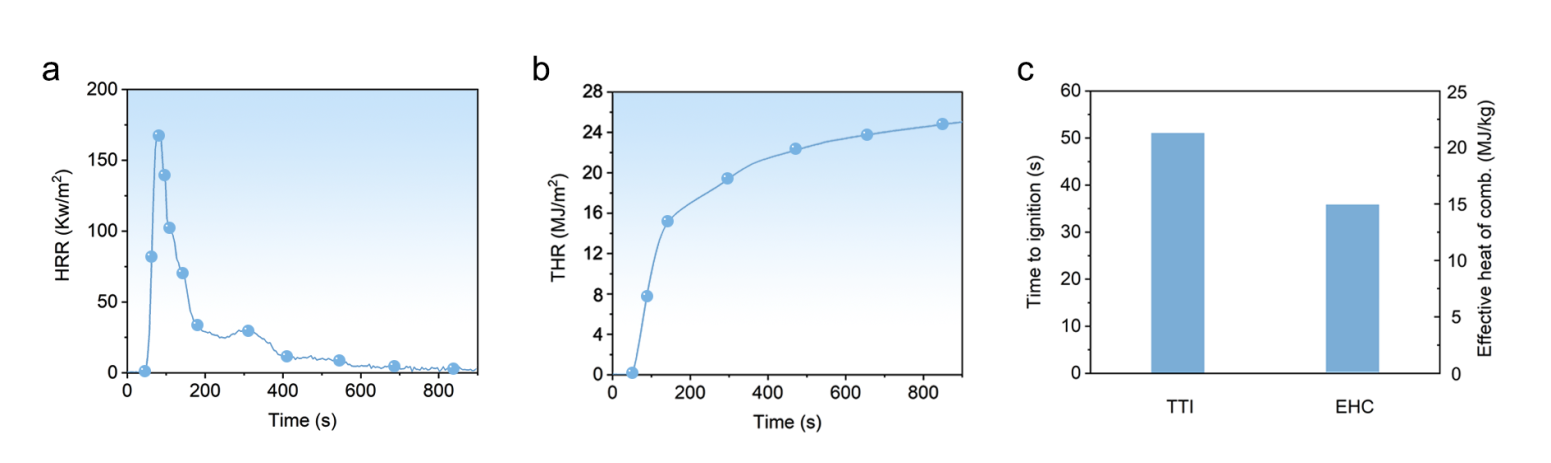


Figure S2. Cone calorimetry results of GLI_15/10_. (a) Heat release rate (HRR), (b) Total heat release (THR), (c) Time to ignition (TTI) and effective heat of combustion (EHC).


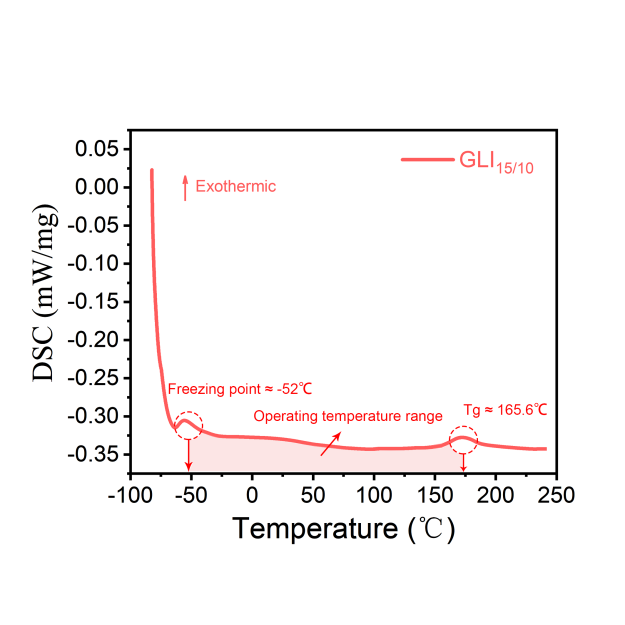


Figure S3. Low-temperature DSC testing.


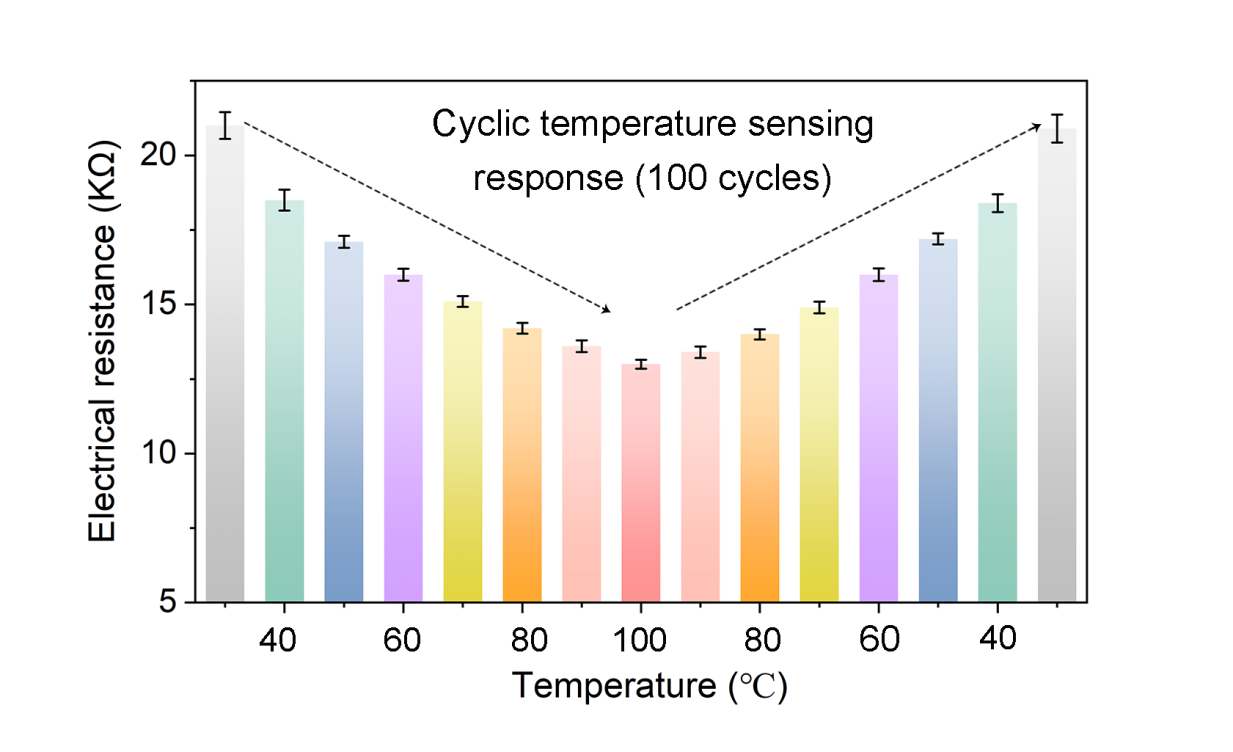


Figure S4. Cyclic temperature sensing response of GLI_15/10_.

*
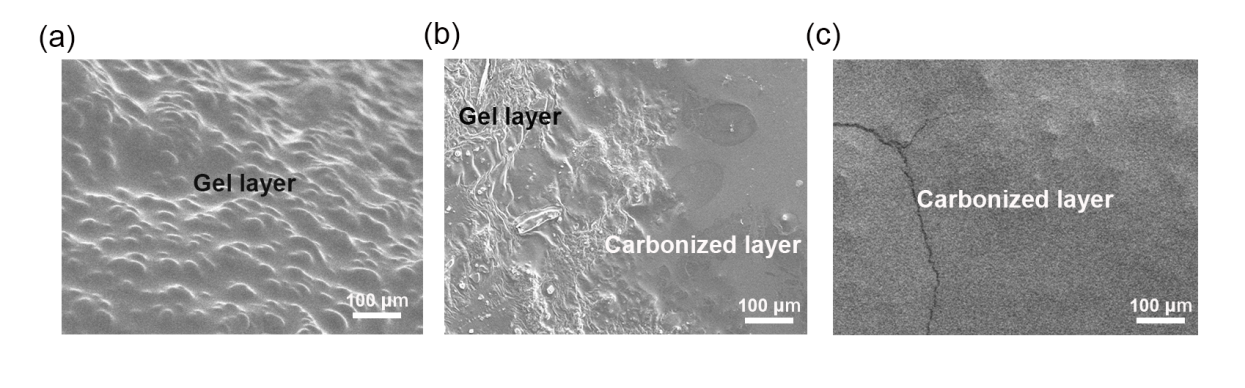
*

Figure S5. SEM of GLI at different stages. (a) Before combustion, (b) during combustion, and (c) after combustion.


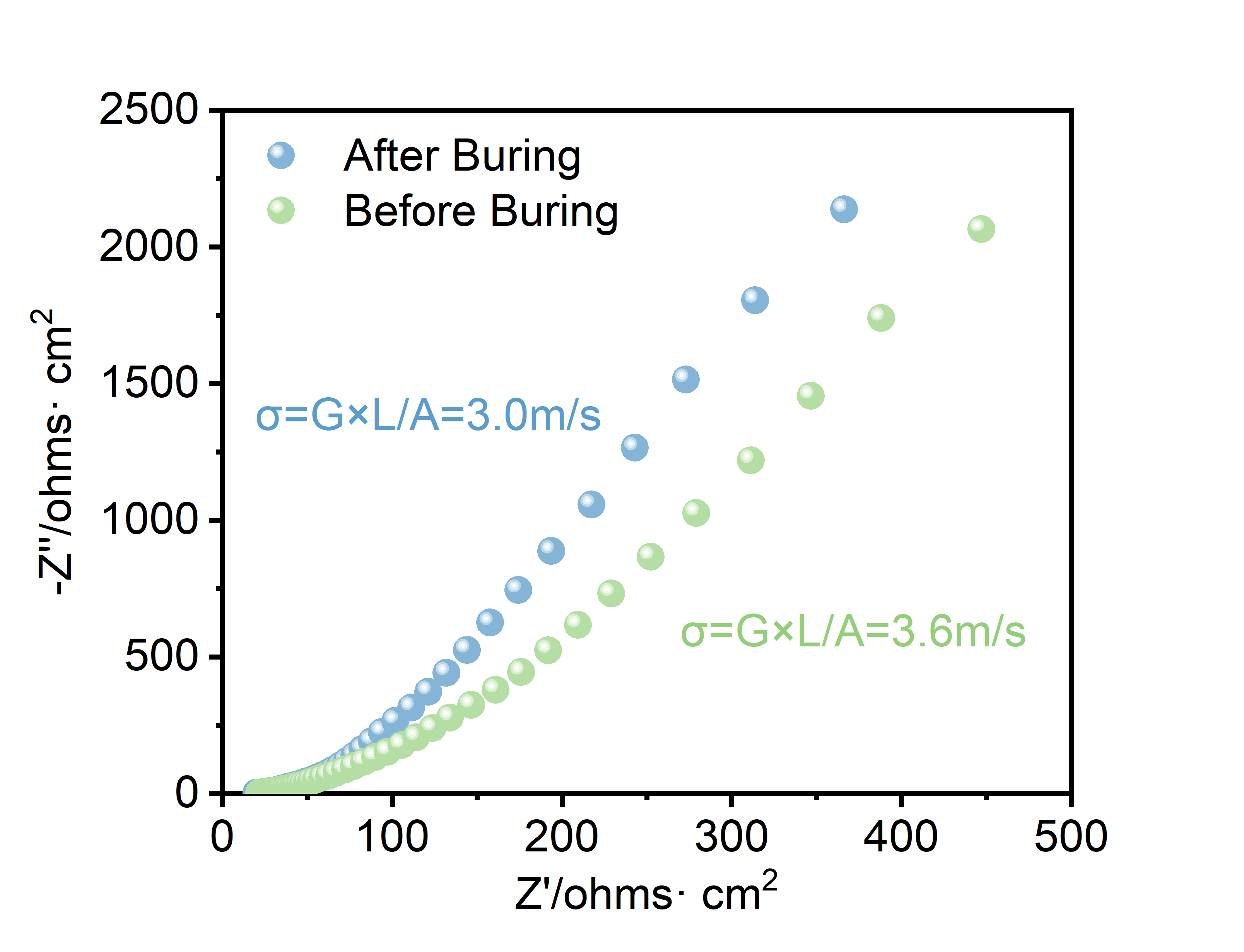


Figure S6. The impedance spectra of GLI before and after combustion.


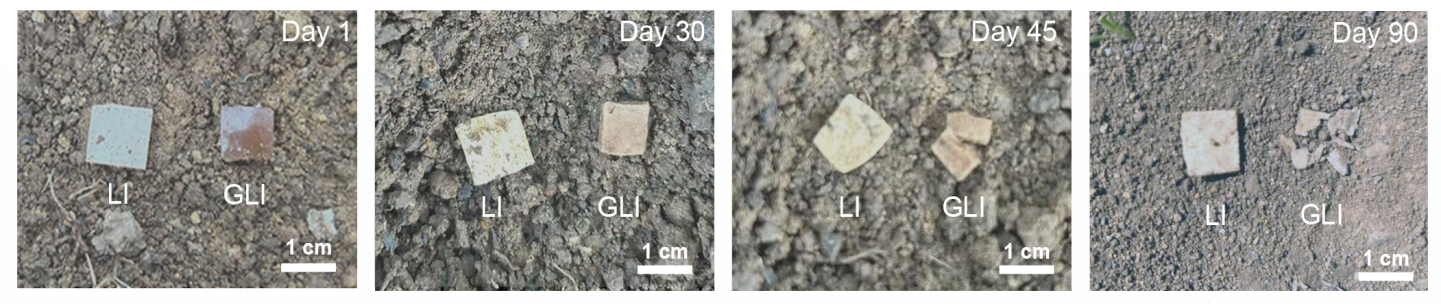


Figure S7. Degradation process under outdoor environmental conditions by 90 days.


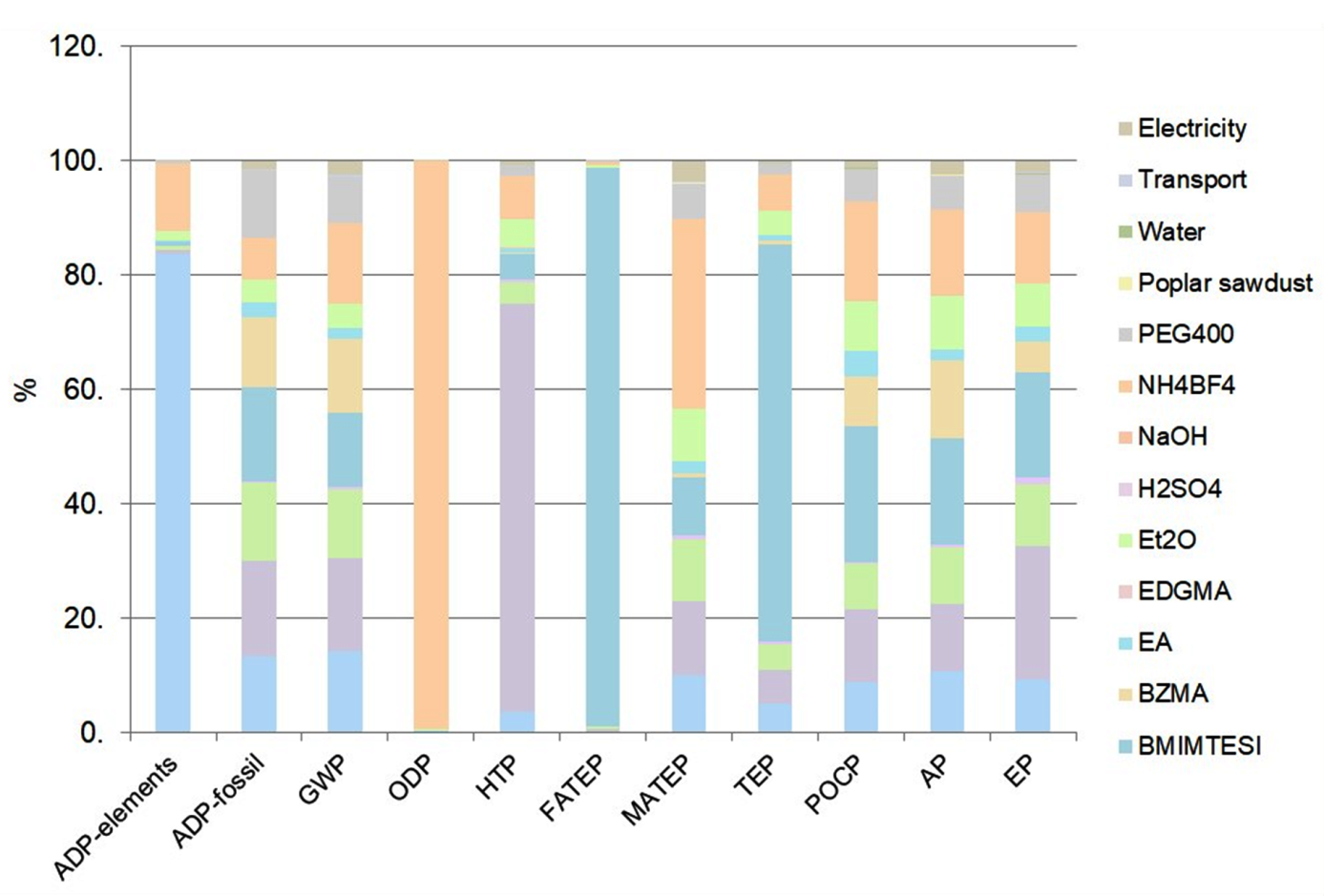


Figure S8. Contribution analysis results per ton GLI_15/10_.


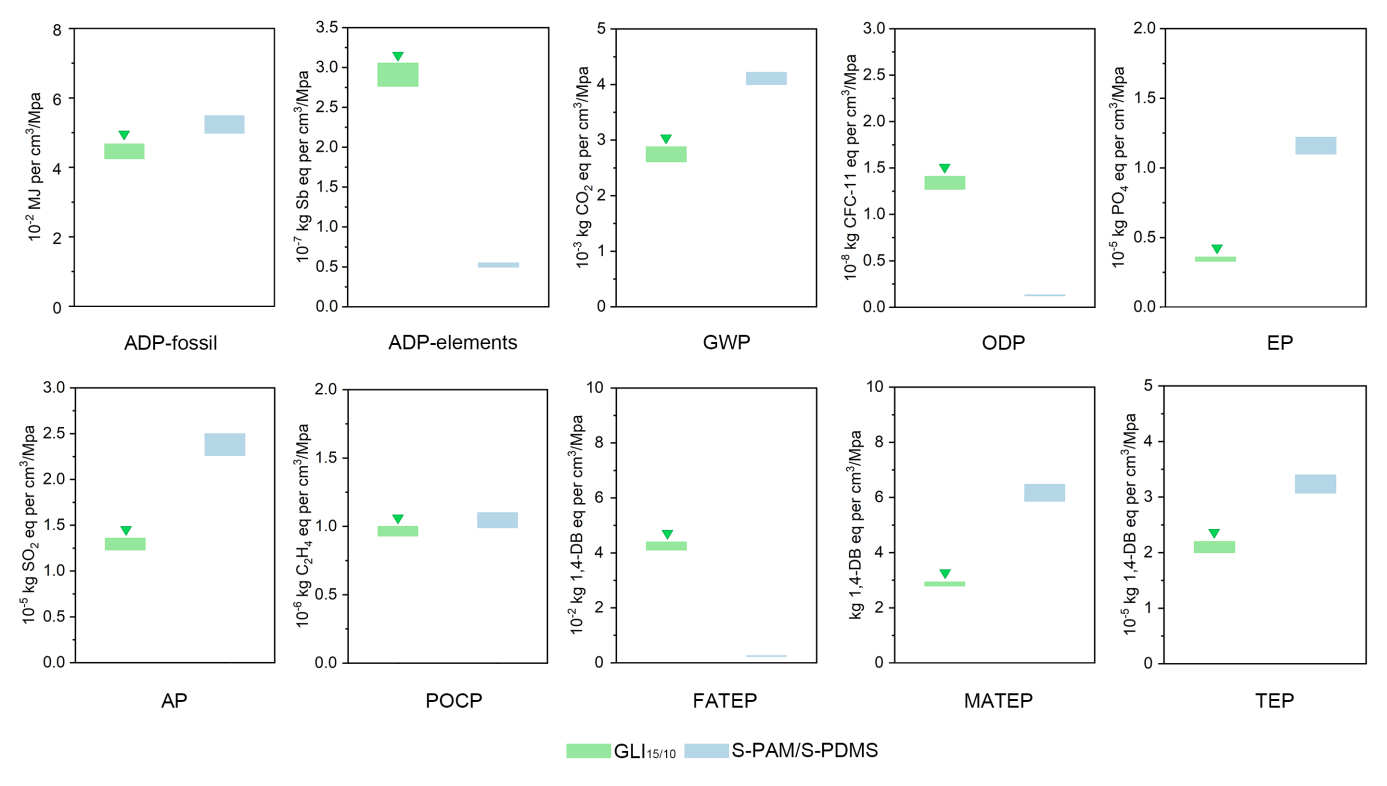


Figure S9. Environmental impact of GLI_15/10_ compared to S-PAM/S-PDMS (per cm^3^/MPa).

**II. Supplementary Tables**

Table S1. Lifecycle inventory data for Gly-lignin ionogel production (1t).

| **Input** | **Amount** | **Unit** | **Source of inventory** |
| --- | --- | --- | --- |
| Poplar sawdust | 0.073 | t | Ecoinvent 3 |
| Sulfuric acid | 0.0029 | t | Ecoinvent 3 |
| Polyethylene glycol 400 | 0.365 | t |  |
| Water | 0.365 | t | Ecoinvent 3 |
| Sodium hydroxide | 0.0058 | t | Ecoinvent 3 |
| Ammonium fluoborate | 0.155 | t |  |
| 1-vinylimidazole | 0.34 | t |  |
| 1-bromobutane | 0.34 | t |  |
| Ether | 0.07 | t | Ecoinvent 3 |
| Ethyl acetate | 0.07 | t | Ecoinvent 3 |
| Benzyl Methacrylate | 0.23 | t |  |
| 1-butyl-3-methylimidazolium bis(trifluoromethyl sulfonyl )imide | 0.35 | t |  |
| 1,4-dioxane | 0.3 | t | Ecoinvent 3 |
| Ethylene glycol diallyl ether | 0.002 | t | Ecoinvent 3 |
| 2,2′-Azodiisobutyronitrile | 0.0035 | t |  |
| Electricity | 300 | kWh | Ecoinvent 3 |
| Transport | 255 | tkm | Ecoinvent 3 |
| **Output** | **Amount** | **Unit** | **Source of inventory** |
| Gly-lignin ionogel | 1 | t |  |
| Cellulose | 0.029 | t | Ecoinvent 3 |
| Hemicellulose | 0.016 | t | Ecoinvent 3 |
| Ammonium bromide | 0.145 | t |  |
| Emission | 0.54 | t |  |

Table S2. LCA results comparison from Gly-lignin ionogel and S-PAM/S-PDMS (1t product).

| **Impact category** | **Unit** | **Gly-lignin ionogel** | **S-PAM/S-PDMS** |
| --- | --- | --- | --- |
| Abiotic depletion | kg Sb eq | 1.29E+00 | 6.10E-02 |
| Abiotic depletion (fossil fuels) | MJ | 1.99E+05 | 8.30E+04 |
| Global warming (GWP100a) | kg CO_2_ eq | 1.23E+04 | 7.65E+03 |
| Ozone layer depletion (ODP) | kg CFC-11 eq | 5.95E-02 | 5.10E-01 |
| Human toxicity | kg 1,4-DB eq | 3.02E+04 | 6.62E+03 |
| Fresh water aquatic ecotox. | kg 1,4-DB eq | 1.96E+05 | 3.28E+03 |
| Marine aquatic ecotoxicity | kg 1,4-DB eq | 1.31E+07 | 1.08E+07 |
| Terrestrial ecotoxicity | kg 1,4-DB eq | 1.88E+02 | 5.06E+01 |
| Photochemical oxidation | kg C_2_H_4_ eq | 4.37E+00 | 2.03E+00 |
| Acidification | kg SO_2_ eq | 5.79E+01 | 4.09E+01 |
| Eutrophication | kg PO_4_--- eq | 1.56E+01 | 1.41E+01 |

Table S3. Environmental impact of Gly-lignin ionogel compared to S-PAM/S-PDMS (per cm^3^/MPa)

| **Impact category** | **Unit** | **Gly-lignin ionogel** | **S-PAM/S-PDMS** |
| --- | --- | --- | --- |
| Abiotic depletion | kg Sb eq | 2.91E-07 | 5.23E-08 |
| Abiotic depletion (fossil fuels) | MJ | 4.46E-02 | 5.24E-02 |
| Global warming (GWP100a) | kg CO_2_ eq | 2.75E-03 | 4.21E-03 |
| Ozone layer depletion (ODP) | kg CFC-11 eq | 1.34E-08 | 1.29E-09 |
| Human toxicity | kg 1,4-DB eq | 6.78E-03 | 4.87E-03 |
| Fresh water aquatic ecotox. | kg 1,4-DB eq | 4.40E-02 | 2.40E-03 |
| Marine aquatic ecotoxicity | kg 1,4-DB eq | 2.94E+00 | 6.17E+00 |
| Terrestrial ecotoxicity | kg 1,4-DB eq | 2.18E-05 | 3.23E-05 |
| Photochemical oxidation | kg C_2_H_4_ eq | 9.82E-07 | 1.05E-06 |
| Acidification | kg SO_2_ eq | 1.30E-05 | 2.38E-05 |
| Eutrophication | kg PO_4_--- eq | 3.51E-06 | 1.15E-05 |
